# Defining Emergency Department Asthma Visits for Public Health Surveillance, North Carolina, 2008–2009

**DOI:** 10.5888/pcd11.130329

**Published:** 2014-06-12

**Authors:** Debbie Travers, Kristen Hassmiller Lich, Steven J. Lippmann, Morris Weinberger, Karin B. Yeatts, Winston Liao, Anna Waller

**Affiliations:** Author Affiliations: Kristen Lich, Steven J. Lippmann, Karin Yeatts, Anna Waller, University of North Carolina, Chapel Hill, North Carolina; Morris Weinberger, University of North Carolina, Chapel Hill, Durham Veteran’s Affairs Medical Center, Durham, North Carolina; Winston Liao, North Carolina COPD Task Force, Cary, North Carolina.

## Abstract

**Introduction:**

When using emergency department (ED) data sets for public health surveillance, a standard approach is needed to define visits attributable to asthma. Asthma can be the first (primary) or a subsequent (2nd through 11th) diagnosis. Our study objective was to develop a definition of ED visits attributable to asthma for public health surveillance. We evaluated the effect of including visits with an asthma diagnosis in primary-only versus subsequent positions.

**Methods:**

The study was a cross-sectional analysis of population-level ED surveillance data. Of the 114 North Carolina EDs eligible to participate in a statewide surveillance system in 2008–2009, we used data from the 111 (97%) that participated during those years. Included were all ED visits with an ICD-9-CM diagnosis code for asthma in any diagnosis position (1 through 11). We formed 11 strata based on the diagnosis position of asthma and described common chief complaint and primary diagnosis categories for each. Prevalence ratios compared each category’s proportion of visits that received either asthma- or cardiac-related procedure codes.

**Results:**

Respiratory diagnoses were most common in records of ED visits in which asthma was the first or second diagnosis, while primary diagnoses of injury and heart disease were more common when asthma appeared in positions 3–11. Asthma-related chief complaints and procedures were most common when asthma was the first or second diagnosis, whereas cardiac procedures were more common in records with asthma in positions 3–11.

**Conclusion:**

ED visits should be defined as asthma-related when asthma is in the first or second diagnosis position.

## Introduction

Asthma is a prevalent chronic disease that is associated with significant morbidity, mortality, and health care use (1,2). Surveillance of population trends in asthma prevalence, use of health care services, and morbidity can support public health efforts to plan for and reduce the consequences of asthma. Surveillance of emergency department (ED) visits is critical to these efforts because patterns of asthma-related ED visits may help states decide where to allocate scarce resources to reduce these often preventable visits.

The availability of ED data for asthma surveillance has expanded from primarily claims-based data and self-report surveys to include electronic health records data. A critical policy-relevant question is how to define an asthma-related ED visit for public health surveillance. Asthma diagnoses may appear in the first (presumed primary) or second (presumed secondary) position or in subsequent diagnosis positions (3 through 11). The estimated number of asthma-related ED visits is affected by which diagnosis positions are used. A common approach in national asthma morbidity surveillance is to use only ED visits with a diagnosis of asthma in the first position (3–7), an approach also used in descriptive studies of acute asthma ED care (8,9). However, this definition may underestimate the number of asthma-related ED visits. Alternatively, attributing an ED visit to asthma if asthma occurs in any position will overestimate its impact. This methodological question, which is analogous to balancing sensitivity and specificity of a diagnostic test, has critical implications for allocating scarce resources to public health initiatives.

The question can be informed by population-based ED data from timely syndromic surveillance systems that include clinical data. Syndromic surveillance systems support public health surveillance through statistical tools and aberration detection methods to investigate disease outbreaks and conditions of public health importance (10). Our objective was to develop an operational definition of asthma-attributable ED visits for public health surveillance. We sought to evaluate the effect of including ED visits with an asthma diagnosis in the first position, as well as in the second, third, or subsequent positions.

## Methods

### Data sources

We used population-based ED visit data from the state public health surveillance system, North Carolina Disease Event Tracking and Epidemiologic Collection Tool (NC DETECT). By legislative mandate, all civilian acute care, hospital-affiliated EDs in North Carolina must submit data to NC DETECT (11) for syndromic surveillance. As of 2008, data on 99.5% of all ED visits state-wide were captured in NC DETECT. These data came from 111 of 114 (97%) EDs in North Carolina. In addition to age, sex, and county of residence, NC DETECT’s ED visit data include discharge diagnoses, procedures, and chief complaints. Race and ethnicity data are not available. Diagnoses are recorded by using International Classification of Diseases, 9th Revision, Clinical Modification (ICD-9-CM); procedure codes use ICD-9-CM or Current Procedural Terminology (CPT) procedure codes (12,13). EDs provide NC DETECT with up to 11 discharge diagnosis codes for each visit. Chief complaints are recorded as a mixture of free text and locally developed or vendor-supplied lists.

ED visit data collected in NC DETECT are routinely captured as part of patient care and hospital administration. Hospitals perform their own medical coding for administrative purposes, and then use standardized health record data formats to securely transmit electronic data streams to a data aggregator. Data are monitored regularly for completeness and valid coding; data quality issues are communicated back to hospitals for resolution (14). Although much effort is put into optimizing the quality of NC DETECT data, the system is ultimately reliant on participating hospitals to capture complete and valid information for their own purposes.

### Procedures

We selected all ED visits from NC DETECT that met the following inclusion criteria: 1) patient resided in North Carolina; 2) visit occurred between January 1, 2008, and December 31, 2009, inclusive; and 3) an ICD-9-CM diagnosis code for asthma (493.xx) was present in at least 1 of the 11 discharge diagnosis positions. NC DETECT does not capture true patient identifiers that allow patient tracking between different EDs, but multiple visits by a patient to the same ED can be linked. However, since our interest was in overall health care use, we counted all asthma ED visits as independent and included repeat visits made by the same patients to the same ED. Duplicate visits for the same date, time, and one-way encrypted patient identifiers were deleted.

We performed several preprocessing steps to clean the data and to standardize and group chief complaints, diagnosis codes, and procedure codes. The text fields for chief complaints contain abbreviations, misspellings, and other nonstandard terms. We used the Emergency Medical Text Processor (University of North Carolina, Chapel Hill, North Carolina, 2004), a validated natural-language processing system that codes raw chief-complaint text entries as standardized terms (15). Next, we used an investigator-developed set of keywords to categorize chief complaint segments from the Emergency Medical Text Processor output into major chief complaint groups based on clinical similarity. For example, the “dyspnea” group includes the keywords: “dyspnea,” “shortness of breath,” “DB,” and “difficulty breathing” (full list of text keywords available from author on request).

ICD-9-CM diagnosis codes were grouped using the Clinical Classification Software (CCS) maintained by the Healthcare Cost and Utilization Project and the Agency for Healthcare Research and Quality (16). The CCS is useful for collapsing the over 14,000 ICD-9-CM diagnosis codes into 295 clinically informative groups. CCS provides good coverage of ICD-9-CM codes encountered in ED data (17).

Approximately 18% of visit entries in our sample included 1 or more procedure codes. Although reporting percentages varied, we received some procedure codes from every hospital and for multiple conditions. Thus, there did not appear to be any systematic reporting bias based on hospital or condition. After examining the most common procedure codes in our sample, we created 2 ad hoc procedure code groups that were specific to either asthma or cardiac conditions. We were conservative in our selection of codes; the procedures chosen for each condition had to be clinically likely to be ordered for that condition and unlikely to be ordered for other conditions. The asthma procedure code group includes procedures involving nebulized medications (9394, 93.94, 94640, 94664). The cardiac group includes codes for 12-lead electrocardiogram, cardiac catheterization, and coronary artery bypass surgery (36.06, 3606, 37.22, 3722, 88.53, 8853, 88.56, 8856, 88.72, 8872, 89.52, 8952, 82550, 82553, 84484, 93005). We chose asthma and cardiac groupings because the corresponding procedure codes were common in NC DETECT and represented distinct clinical problems.

Visits were categorized into 11 strata based on the position of the asthma diagnosis code. For example, if an asthma diagnosis code appeared in the fourth listed diagnosis position, the visit would be categorized into stratum 4. Subsequent analyses were stratified by these 11 diagnosis positions.

### Analysis

All analyses were performed separately for children (<18 y) and adults (≥ 18 y). Descriptive analysis included the number and percentage of asthma-related ED visits in each of the 11 diagnosis strata. We then analyzed the most frequent first-listed diagnosis codes, grouped according to the CCS, when asthma first appears in the second, third, or fourth through eleventh diagnosis position. Next, we examined the frequency of the most common chief complaint keyword groups reported for visits in each diagnosis position group. We then assessed the proportion of visits in each group that reported either asthma- or cardiac-related procedure codes and used a linear risk regression model to calculate the prevalence differences and 95% confidence intervals by comparing the prevalence in each diagnosis group with that in the group for asthma in the first diagnosis position. Finally, we examined the most frequent second-listed CCS diagnosis groups when asthma appeared in the first diagnosis position. For the procedure analyses only, we restricted our sample to visits that had at least one procedure code.

This study was reviewed and approved by the Public Health–Nursing Institutional Review Board at the University of North Carolina at Chapel Hill.

## Results

Of the 8.7 million ED visits reported to NC DETECT in 2008 and 2009, 350,341 (4%) resulted in a diagnosis of asthma in one or more of the 11 diagnosis positions (Table 1). Of the visits with an asthma diagnostic code, 29% were made by children (<18 y). Records of ED visits with at least one asthma diagnosis were likely to have multiple diagnosis codes; 58.0% of child visits and 83.9% of adult visits with asthma codes were assigned 3 or more total diagnosis codes, compared with 52.4% of all ED visits. Overall, asthma appears in the first-listed position for 36,170 (35.9%) child visits and 59,572 (23.9%) adult visits (Table 1). Including both the first and second listed asthma diagnosis positions nearly doubles this to 67,317 (66.7%) child visits and 111,358 (44.7%) adult visits. Two-thirds of child visits had asthma diagnoses in the first 2 positions, whereas adult visits with asthma diagnoses were spread more widely across the 11 positions.

**Table 1:** Diagnosis Position in Which Asthma Was First Recorded, North Carolina Emergency Departments (EDs), 2008–09

Diagnosis Position Where Asthma First Appears	Number of Visits with an Asthma Diagnosis	Percentage Asthma visits	Cumulative Percentage	Percentage all ED visits^a^^,^ b	Cumulative % all ED visits^a^
1	95,756	27.3	27.3	1.10	1.10
2	82,953	23.7	51.0	0.95	2.06
3	61,537	17.6	68.6	0.71	2.77
4	40,272	11.5	80.1	0.46	3.23
5	26,030	7.4	87.5	0.30	3.53
6-11	43,793	12.4	100	0.51	4.03
Total	350,341	—			
**Children (0–17 y)**					
1	36,170	35.85	**—**		
2	31,147	30.87	**—**		
3	18,847	18.68	**—**		
4	8,400	8.33	**—**		
5	3,673	3.64	**—**		
6-11	2,649	2.62	**—**		
Total	100,886 (29%)^c^	—	—		
**Adults (≥18 y)**					
1	59,572	23.89	**—**		
2	51,786	20.76	**—**		
3	42,683	17.11	**—**		
4	31,867	12.78	**—**		
5	22,354	8.96	**—**		
6-11	41,141	16.48	**—**		
**Total**	**249,403** (71%)^c^	—	**—**		

Abbreviation: —, not applicable.

a All ED visits total approximately 8.7 million.

b Age-specific numbers may not sum to the total; age variable missing for 52 (0.02%) of visits.

c Percentage of 350,341, total number of asthma visits.

**Figure 1:**
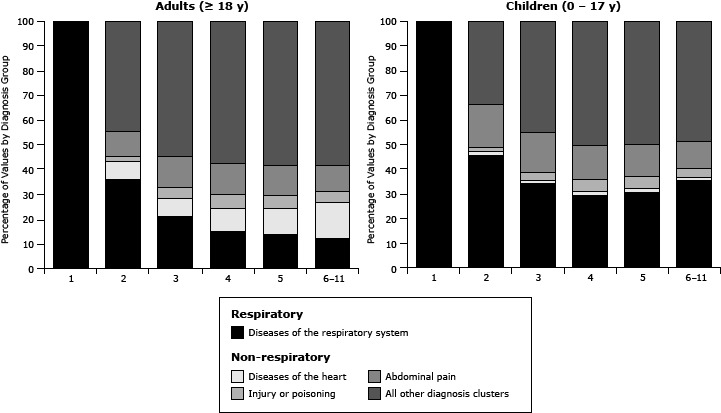
Grouped primary diagnosis by asthma diagnosis position, North Carolina emergency department visits, 2008–09, with an asthma diagnosis in any diagnosis position (child visits, N = 100,886; adult visits, N = 249,403). **Table:** 

**Grouped diagnoses:** Figure 1 illustrates the most frequent primary ICD-9-CM diagnosis groupings by diagnosis position in which the asthma diagnosis first appears. For children, respiratory diagnoses (eg, influenza, pneumonia) are the most common reason for ED visits during which asthma was assigned to the second diagnosis position, whereas other diagnoses are most common for adults with asthma in the second position. For both children and adults, the proportion of respiratory visits gradually drops to positions 3–11. In particular for adults, primary diagnoses of heart disease are more common in records when asthma appears in positions 3–11.

Diagnosis Group	Position of Asthma Diagnosis					
	1	2	3	4	5	6–11
**Children (0–17 y), %**						
**Diseases of the** **respiratory system**	100	46	34	30	31	35
**Diseases of the heart**	0	1	2	2	2	2
**Abdominal pain**	0	2	3	5	4	3
**Injury or poisoning**	0	18	16	13	13	11
**All other diagnosis clusters**	0	33	45	50	50	49
**Adults (≥18 y), %**						
**Diseases of the** **respiratory system**	100	36	21	15	14	13
**Diseases of the heart**	0	7	8	9	11	14
**Abdominal pain**	0	10	12	12	11	10
**Injury or poisoning**	0	2	5	6	5	5
**All other diagnosis clusters**	0	44	55	58	59	58

**Grouped chief complaints:** The chief complaint data followed a similar pattern: respiratory chief complaints are most common in visits in which asthma was assigned in the first (63%, children; 57%, adults) and second (32% children; 28%, adults) diagnosis positions. Nonrespiratory chief complaints were increasingly prevalent when asthma was in positions 3 through 11, reaching 90% for adults and 84% for children in positions 6 through 11 combined.

**Figure 2:**
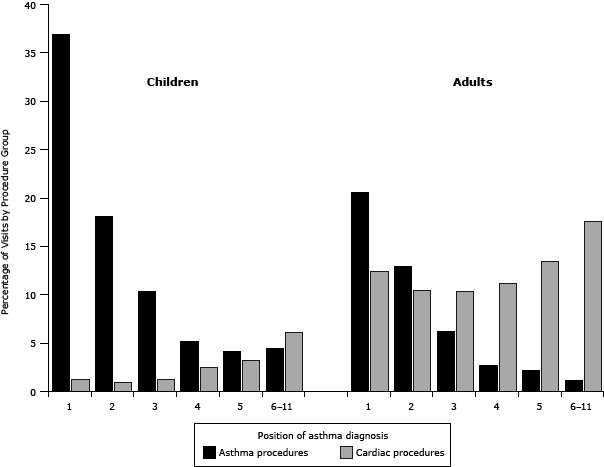
Grouped respiratory and cardiac procedures by asthma diagnosis position, North Carolina emergency department visits, 2008–09, with an asthma diagnosis in any diagnosis position and 1 or more procedure codes assigned (child visits, N = 13,125; adult visits, N = 49,265). **Table:** 

**Grouped procedures:** Of the 350,341 visits with asthma diagnoses, 62,399 (18%) had 1 or more procedure codes assigned, which was similar to the percentage in the overall NC DETECT data. Of visits by children, 13,125 (13%) had one or more procedures compared with 49,265 (20%) of visits by adults. Figure 2 illustrates the percentage of visits that had asthma versus cardiac procedures by age group. For both groups, asthma procedure codes were most commonly assigned to visits with asthma in the first or second diagnosis position. Cardiac procedures were increasingly common in records with asthma in positions 3 through 11, particularly in adults. For children, the prevalence of asthma-related procedures decreased from 36.8% of visits with asthma in the first diagnosis position to 18.1% and 10.3% when asthma was in the second or third positions, respectively (Table 2). For adults, the prevalence of asthma-related procedures was lower but followed a similar pattern. Conversely, cardiac procedure prevalence for adults increased substantially with lower asthma diagnosis rank, from 12.4% with asthma in the first position to 17.6% with asthma in positions 6 through 11. Prevalence differences calculated for both children and adults for the asthma and cardiac procedures showed that these changes in prevalence were significant (Table 2).

Procedure Group	1	2	3	4	5	6-11
**Children (0–17 years), %**						
**Asthma**	37	18	10	5	4	4
**Cardiac**	1	1	1	2	3	6
**Adults (≥18 years), %**						
**Asthma**	21	13	6	3	2	1
**Cardiac**	12	10	10	11	13	18

**Table 2:** Proportion of Emergency Department (ED) Visits with Asthma-Related^a^ and Cardiac-Related^b^ Procedure Codes, by Asthma Diagnosis Position, Among ED Visits With At Least One Procedure Code Entered^c^, North Carolina EDs, 2008–09

Position of asthma diagnosis	Total ED Visits with at Least 1 Procedure Code Recorded	Asthma-Related Procedure N (%)	Prevalence Ratio for Asthma-Related Procedures (95% CI)	Prevalence Difference for Asthma-Related Procedures (95% CI)	Cardiac-Related Procedure N (%)	Prevalence Ratio for Cardiac-Related Procedures (95% CI)	Prevalence Difference for Cardiac-Related Procedures (95% CI)
**All Ages**							
1	10,790	2,729 (25.3)	1 [Reference]	1 [Reference]	987 (9.1)	1 [Reference]	1 [Reference]
2	14,547	2,118 (14.6)	0.58 (0.55– 0.61)	−10.7 (−11.7– −9.7)	1–074 (7.4)	0.81 (0.74– 0.88)	−1.8 (−2.5– −1.1)
3	11,309	823 (7.3)	0.29 (0.27– 0.31)	−18 (−19– −17.1)	896 (7.9)	0.87 (0.79– 0.94)	−1.2 (−2– −0.5)
4	8,145	252 (3.1)	0.12 (0.11– 0.14)	−22.2 (−23.1– −21.3)	790 (9.7)	1.06 (0.97– 1.16)	0.6 (−0.3– 1.4)
5	5,632	131 (2.3)	0.09 (0.08– 0.11)	−23 (−23.9– −22.1)	699 (12.4)	1.36 (1.24– 1.49)	3.3 (2.3– 4.3)
6-11	11,976	151 (1.3)	0.05 (0.04– 0.06)	−24.0 (−24.9– −23.2)	2,049 (17.1)	1.87 (1.74– 2.01)	8.0 (7.1– 8.8)
**Total**	**62,399**	**6,204 (9.9)**	—	—	**6,495 (10.4)**	—	—
**Children (0–17 years)**							
1	3,121	1,150 (36.8)	1 [Reference]	1 [Reference]	39 (1.2)	1 [Reference]	1 [Reference]
2	4,650	843 (18.1)	0.49 (0.46– 0.53)	−18.7 (−20.7– −16.7)	45 (1.0)	0.77 (0.51– 1.19)	−0.28 (−0.8– 0.2)
3	2,966	306 (10.3)	0.28 (0.25– 0.31)	−26.5 (−28.6– −24.5)	37 (1.2)	1.00 (0.64– 1.56)	0 (−0.6– 0.6)
4	1,348	70 (5.2)	0.14 (0.11– 0.18)	−31.7 (−33.7– −29.6)	33 (2.4)	1.96(1.24– 3.10)	1.2 (0.3– 2.1)
5	563	23 (4.1)	0.11(0.07– 0.17)	−32.8 (−35.1– −30.4)	18 (3.2)	2.56 (1.47– 4.44)	2.0 (0.4– 3.5)
6-11	477	21 (4.4)	0.12 (0.08– 0.18)	−32.4 (−35.0– −29.9)	29 (6.1)	4.87 (3.04– 7.79)	4.8(2.7– 7.0)
**Total**	**13,125**	**2,413 (18.4)**	—	—	**201 (1.5)**	—	—
**Adults (≥18 years)**							
1	7,667	1579 (20.6)	1 [Reference]	1 [Reference]	948 (12.4)	1 [Reference]	1 [Reference]
2	9,893	1275 (12.9)	0.63 (0.58– 0.67)	−7.7 (−8.8– −6.6)	1,029 (10.4)	0.84 (0.77– 0.91)	−2.0 (−2.9– −1.0)
3	8,343	517 (6.2)	0.30 (0.27– 0.33)	−14.4 (−15.4– −13.4)	859 (10.3)	0.83 (0.76– 0.91)	−2.1 (−3.1– −1.1)
4	6,796	182 (2.7)	0.13 (0.11– 0.15)	−17.9 (−18.9– −16.9)	757 (11.1)	0.90 (0.82– 0.99)	−1.2 (−2.3– −0.2)
5	5,067	108 (2.1)	0.10 (0.09– 0.13)	−18.5 (−19.5– −17.5)	681 (13.4)	1.09 (0.99– 1.19)	1.1 (−0.1– 2.3)
6-11	11,499	130 (1.1)	0.05 (0.05– 0.07)	−19.5 (−20.4– −18.5)	2,020 (17.6)	1.42 (1.32– 1.53)	5.2 (4.2– 6.2)
**Total**	**49,265**	**3,791 (7.7)**	—	—	**5,612 (11.4)**	—	—

Abbreviations: CI, confidence interval; —, not applicable.

a Asthma procedures: 9394, 93.94, 94640, 94664. These procedure codes represent nebulization treatment (93.94 and 94640) or demonstration of a nebulizer or inhaler (94664). Entries in the NC DETECT data are not standardized; some contained decimals and some did not.

b Cardiac procedures: 36.06, 3606, 37.22, 3722, 88.53, 8853, 88.56, 8856, 88.72, 8872, 89.52, 8952, 82550, 82553, 84484, 93005. These procedure codes represent cardiac ultrasonography, electrocardiogram (12 leads), 2 catheter coronary arteriogram, left heart angiocardiogram, left heart cardiac catheterization, creatinine kinase (total and fraction only), troponin (quantitative), and insertion of coronary artery stent.

c Restricted to visits that had at least 1 procedure code entered; those that had only nonspecific Evaluation and Management codes for emergency department visits (99281, 99282, 99283, 99284, 99285) were excluded.

For visits with asthma in the first diagnosis position, we identified the second diagnosis. When asthma was in the first position, the most common second listed diagnosis was some type of respiratory disease or infection including other lower respiratory disease, other upper respiratory infection, acute bronchitis, or pneumonia.

## Discussion

The syndromic surveillance community has recognized the need for developing standardized operational definitions to facilitate comparisons across surveillance systems. An NIH-funded consensus conference developed standard definitions for 4 acute infectious diseases of public health importance (18). Because syndromic surveillance data are also used to monitor chronic conditions such as asthma, a similar standardization is needed. In addition to public health, core measures for digital data, including electronic health records, are needed for clinical research (19). Our study addresses this issue by proposing a definition of ED visits attributable to asthma for public health surveillance.

Specificity is maximized by limiting asthma-only visits with asthma in the first (presumed primary) diagnosis position; however, our findings suggest doing so will underrepresent the burden of asthma morbidity. Instead, for public health surveillance, the grouped diagnosis and chief complaint data suggest including asthma in the first or second diagnosis position will capture visits that are appropriately attributed to asthma; the procedure data also suggest this, but were available from NC DETECT for only 18% of the visits.

Consider this realistic example of diagnosis coding of 3 ED patient records with asthma ICD-9-CM codes in various diagnosis positions:

Patient A: 1) 493.02, asthma with acute exacerbation; 2) 796.2, elevated blood pressure
Patient B: 1) 465, acute upper respiratory infection; 2) 493.02, asthma with acute exacerbation
Patient C: 1) 928.20, crushing injury, foot; 2) 924.2; contusion, ankle; 3) 493.9, asthma unspecified.

If all 3 patient visits were attributed to asthma, the surveillance system would be very sensitive but would lack specificity. However, if the prevailing approach was used and only Patient A was included in the surveillance system, false positives would be reduced but sensitivity would suffer. Including only Patients A and B might provide an optimal balance between sensitivity and specificity. Here, Patient B had an upper respiratory infection in the first listed diagnosis position. It is likely that this infection triggered the asthma exacerbation; therefore, this is appropriately classified as an asthma ED visit.

Without a standard definition of an ED asthma visit, a marked difference exists in the number of ED visits attributed to asthma depending on which diagnosis positions are used. Including in our data ED visits with asthma in the second diagnosis position nearly doubled the estimated number of ED visits related to asthma. Although adding ED visits with asthma in the third position would substantially increase the number of asthma-related ED visits, our analyses of chief complaints and procedures suggest a nonasthma-related reason for the ED visit. Because the procedure data suggest that some visits with asthma in the third position might be attributed to asthma, further study using algorithmic approaches may be useful in reaching a more accurate asthma definition. For example, a visit with asthma in the second or third ED diagnosis position might be classified as attributable to asthma if it also included a respiratory chief complaint and procedure plus a first-listed diagnosis relevant to asthma exacerbations. A similar approach was explored with claims data from managed care and state public assistance sources to support pediatric asthma surveillance in a community in California (20). Those researchers developed a definition for ED, outpatient, or hospital visits attributable to asthma that included visits with asthma in the first or second position. Visits with asthma in the second position were considered attributable to asthma if the first listed diagnosis was a condition commonly precipitated by an asthma exacerbation (eg, pneumonia, respiratory failure). Our study and their study (20) indicate the need for further research validating which primary nonasthma diagnoses are highly associated with true asthma visits. Our findings also suggest differences in patterns of diagnoses and procedures for children that should be explored further.

A trade-off will always exist between sensitivity and specificity in syndromic surveillance systems that use secondary data, and the final decision should be determined on the basis of the specific purpose of a particular study. The goal of having a public health surveillance definition for asthma-related ED visits is to capture most accurately the burden of asthma for public health planning and action. Akinbami et al. (2) suggested that a narrower definition is more appropriate for clinical purposes, but a broader definition is useful for examining health care use or disparities among patients with asthma. Clinical research and clinical decision-making (such as those used in triage algorithms and decision support) may require the greater specificity of a narrower definition. However, we believe that public health surveillance requires a broader definition. Based on the results of this and our previous study (21), the NC DETECT system now defines ED visits attributable to asthma as those with an asthma diagnosis in the first or second position. This approach allows public health surveillance professionals to improve their monitoring of asthma ED visits at the state and local levels. Epidemiologists from the NC Division of Public Health participated in the development and implementation of this definition and are active users of the NC DETECT system.

Limitations of this study are inherent to secondary data, such as coding variability and accuracy. Procedure data were available for only 18% of the visits. EDs are instructed to send ED records to NC DETECT with the primary diagnosis in position 1; however, that the EDS have done as instructed is difficult to validate (14), although our unpublished analysis supports this assumption. In addition, there was no gold standard for an ED visit attributable to asthma, so we could not calculate sensitivity, specificity, and positive and negative predictive value. We recommend further testing of this proposed definition through comparisons with a gold standard (manual record review). Finally, the study was conducted in North Carolina and may not be generalizable to other regions of the country.

This population-based study suggests that operational definitions used in public health surveillance have critical implications for allocating scarce resources to public health initiatives. Although asthma-attributable ED visits are often defined by using only the first diagnosis code, extending the definition to the second nearly doubles our estimate of asthma-attributable ED use. Our study suggests that expanding the traditional definition seems warranted on the basis of analysis of co-reported diagnoses, chief complaints, and procedures. This and similar studies focused on other disease conditions or populations are increasingly possible for public health surveillance as electronic health records include more clinical data and informatics tools such as natural language processing systems become available for extracting chief complaints and clinical notes from electronic health records.
